# Association of patient experience and the quality of hospital care

**DOI:** 10.1093/intqhc/mzad047

**Published:** 2023-07-03

**Authors:** Rawia Abdalla, Milena Pavlova, Wim Groot

**Affiliations:** Department of Health Services Research, CAPHRI, Maastricht University Medical Center, Faculty of Health, Medicine and Life Sciences, Maastricht University, P.O. Box 616, Maastricht, Limburg 6200 MD, The Netherlands; Department of Health Services Research, CAPHRI, Maastricht University Medical Center, Faculty of Health, Medicine and Life Sciences, Maastricht University, P.O. Box 616, Maastricht, Limburg 6200 MD, The Netherlands; Department of Health Services Research, CAPHRI, Maastricht University Medical Center, Faculty of Health, Medicine and Life Sciences, Maastricht University, P.O. Box 616, Maastricht, Limburg 6200 MD, The Netherlands; Top Institute Evidence-Based Education Research (TIER), Maastricht University, P.O. Box 616, Maastricht, Limburg 6200 MD, The Netherlands

**Keywords:** patient experience, patient-reported experience measures, clinical outcomes, clinical quality, quality of care, value-based health care

## Abstract

The association between patient experience and the quality of hospital care is controversial. We assess the association between clinical outcomes and patient-reported experience measures (PREMs) in hospitals in Saudi Arabia. Knowledge on this issue informs value-based health-care reforms. A retrospective observational study was conducted in 17 hospitals in Saudi Arabia during the period of 2019–22. Hospital data were collected on PREMs, mortality, readmission, length of stay (LOS), central line–associated bloodstream infection (CLABSI), catheter-associated urinary tract infection (CAUTI), and surgical site infection. Descriptive analysis was used to describe hospital characteristics. Spearman’s rho correlation tests were used to assess the correlation between these measures, and multivariate generalized linear mixed model regression analysis was used to study associations while controlling for hospital characteristics and year. Our analysis showed that PREMs were negatively correlated with hospital readmission rate (*r* = −0.332, *P *≤ .01), LOS (*r* = −0.299, *P *≤ .01), CLABSI (*r* = −0.297, *P *≤ .01), CAUTI (*r* = −0.393, *P *≤ .01), and surgical site infection (*r* = −0.298, *P *≤ .01). The results indicated that CAUTI and LOS converged negatively with PREMs (*β* = −0.548, *P *= .005; *β* = −0.873, *P *= .008, respectively) and that larger hospitals tended to have better patient experience scores (*β* =0.009, *P *= .003). Our findings suggest that better performance in clinical outcomes is associated with higher PREM scores. PREMs are not a substitute or surrogate for clinical quality. Yet, PREMs are complementary to other objective measures of patient-reported outcomes, the process of care, and clinical outcomes.

## Introduction

Patient experience, defined as the range of interactions that patients have with the health-care systems, including care providers and health-care facilities, is increasingly gaining attention as one of the key parameters used to drive quality improvement in health-care services [1]. This is part of a larger endeavour to transform traditional health-care systems into more patient-centred and value-based care delivery systems. On the one hand, patient-centred care aims to improve the sensitivity and responsiveness to individual patient preferences, needs, and values and to ensure that patient values guide clinical decisions [2]. On the other hand, value-based health care (VBHC) aims to achieve the best health outcomes at the lowest cost to optimize value for patients [3]. These two approaches (patient-centredness and VBHC) overlap in prioritizing patient perspectives and outcomes for health-care reform and emphasize what patients value. Accordingly, key initiatives, such as the six aims for the improvement of health-care systems from the Institute of Medicine and the Triple Aim Framework of the Institute for Healthcare Improvement, have included patient experience as an integral component in their framework for improving health-care services [4, 5].

Related to the VBHC context, VBHC programmes foster the integration of patient-reported experience measures (PREMs) into quality measurement systems in conjunction with clinical outcomes and patient-reported outcome measures [6]. Although PREMs have been integrated into public reporting programmes in many countries, controversy exists on PREMs’ association with clinical quality of care. A large body of literature has reported a positive association between PREMs and clinical outcomes [7–17]. However, other publications have reported no association [18–24]. Methodological issues such as measurement practices, sample size, and survey tools may contribute to this mixed evidence and raise concerns about the relation between PREMs and clinical outcomes, and to what extent PREMs can reflect these measures. A summary of the relevant literature is provided in Supplementary File.

Despite these efforts to study the association between PREMs and other measures of value, no published evidence was found on this association in Saudi Arabia. In line with the Saudi national transformation programme and the 2030 vision that sets the implementation of VBHC as one of its major goals, the Saudi Patient Experience Centre has established the Patient Experience Measurement programme with different outcomes that mainly include developing, comparing, and improving patient experience at the national and international levels [25]. Some studies have measured patient satisfaction and experience in different Saudi settings, such as primary care [26, 27], emergency [28], and oncology [29]. The latest patient experience reports show that 79% of Saudi patients are satisfied with their experience in health-care facilities [25]. However, these results were not linked to any improvement in quality measures, and the association between PREMs and clinical outcomes was not tested before in the country.

Therefore, our research question was as follows: do hospitals that provide a better quality of care in terms of improved clinical outcomes have better PREM scores? Our aim was to assess the association between clinical outcomes and PREMs in hospitals in Saudi Arabia to inform VBHC reforms. This is the first study that examines the current relationship of PREMs with other quality measures in Saudi Arabia and can be used as a reference to different patient experience–based quality improvement initiatives in the region. This study is also relevant to other countries that aim to implement VBHC by endorsing measures that show an association between PREMs and clinical quality, so efforts can be mounted to improve these measures that can have a higher impact on value compared to other discrete measures.

## Methods

### Study design and setting

A retrospective observational study to examine the association between PREMs and selected clinical outcomes was conducted during the period of 2019–22 in 17 hospitals from different geographical regions across Saudi Arabia. Ethical and hospital approvals were obtained from the participating hospitals to collect data related to the study’s measures (ethical approval numbers: ECC# 2022-11 and FHML-REC/2021/116).

### Data collection

Purposive sampling was applied to select hospitals that used the Press Ganey survey to collect data on patient experience and measured PREMs, hospital mortality, readmission, length of stay (LOS), and hospital-acquired infections (HAIs), including central line–associated bloodstream infections (CLABSI), catheter-associated urinary tract infections (CAUTI), and surgical site infections (SSIs) from the first quarter 2019 until the end of the first quarter 2022 (13 quarters). Data collection and measurement procedures for all clinical outcomes were standardized prior to including the hospital in the study to ensure that the measure’s specifications, such as definition and calculation, were consistent in all hospitals. The participating hospitals were requested to provide retrospective quarterly reports of readily available data on PREMs and the selected clinical outcomes for the study period. As the aim of this study was to assess if better hospital performance in clinical quality is associated with better PREMs, data were collected and analysed at the hospital level, not at the individual patient level. Although the patients treated in the hospital may change, the focus of this study was directed to the overall hospital practices and quality improvement initiatives that may change over time and affect the outcomes of individual patients. Multiple measurements of hospital performance at different time points were collected as repeated measures of hospital-level quality indicators. Accordingly, data collection included quality measures that were repeatedly measured in each of the 17 involved hospitals over 13 consecutive quarters. This resulted in a longitudinal dataset that included 221 repeated measures for all involved hospitals. In addition, the characteristics of the included hospitals that could influence the association between PREMs and clinical outcomes, such as hospital size, location (urban/rural), ownership (public/private), teaching status (teaching/nonteaching), accreditation status (accredited/non-accredited), and hospital affiliation (part of hospital network/stand-alone hospital), were collected for risk-adjustment purposes [7–9, 12, 22].

### Patient-reported experience measures

PREMs in the included hospitals were measured using the in-patient satisfaction survey conducted by Health Links, the regional partner of Press Ganey. This survey was validated in Saudi Arabia with an overall Cronbach’s alpha of 0.915 and a range from 0.739 to 0.931 for the domains [30]. It consists of 34 questions classified into 10 domains, including the admission process, speed of admission, room, meals, nurses, doctors, personal issues, visitors and family, discharge, and overall assessment [30]. Two hospitals used satisfaction surveys that measured the same domains of the Press Ganey survey in addition to other measures of patient satisfaction. For the purpose of this study, the overall rating of PREMs was considered, and accordingly, the participating hospitals were requested to provide a retrospective quarterly report of PREMs’ overall ratings for the study period.

### Clinical outcomes

The clinical outcomes were selected based on a literature review of the relevant studies that investigated the association between PREMs and clinical outcomes [7–24], subject matter consultation, and availability and feasibility of data in the included hospitals. These clinical outcomes included hospital mortality, readmission, LOS, CLABSI, CAUTI, and SSI. Furthermore, these clinical outcomes are part of value-based and pay-for-performance programmes, and their measurement is assumed to be common, imperative, and fundamental practice to evaluate the quality of care in hospitals. The hospitals were requested to provide a retrospective quarterly report on these clinical outcomes for the study period.

### Statistical analysis

Our analysis used repeated measures of the seven variables included in this study (namely PREMs, mortality, readmission, LOS, CLABSI, CAUTI, and SSI) measured as rates or percentages. Descriptive analysis was used to describe these continuous variables, including means and standard deviations. Hospital characteristics, as categorical independent variables, were described using frequencies and percentages. The Kolmogorov–Smirnov test of statistical normality was used to assess the statistical normality assumption for the continuous variables. The wide format dataset was used in the descriptive analysis and then restructured into the long format dataset of repeated measures (i.e. longitudinal data) to account for time points as a vector in the rest of the analyses. Spearman’s rho correlation test was used to assess the correlation between PREMs, clinical outcomes, and hospital characteristics. Block-wise multivariate generalized linear mixed model regression analysis was done to assess the association between hospital characteristics and these quality measures expressed as *β* coefficients with 95% confidence intervals. We used a forward block-by-block modelling method where each block consisted of a set of predictors. The selection of the predictors in each block was informed by our prior knowledge gained from the comprehensive literature review on the predictors that were expected to have related effects on PREMs, as dependent variables, and from the correlation analysis to produce more meaningful models for analysis. The predictors and analysis terms in each model were manually specified by the research team in the Statistical Package for Social Sciences (SPSS) program. The analysis resulted in many competing models. The model with the greatest goodness-of-fit in terms of lower Akaike information criterion (AIC) and Bayesian information criterion (BIC) was preferred. Version 28 of the SPSS IBM statistical analysis program was used for the statistical analysis.

## Results

A total of 17 hospitals in Saudi Arabia participated in the study. Each of these hospitals had 13 observations measured in 13 consecutive quarters starting from the first quarter of 2019 to the end of the first quarter of 2022. Accordingly, the data matrix included 221 observations for the 17 hospitals over 13 quarters. The findings showed that 70.6% (*n* = 12) of the hospitals were nonteaching and in urban areas, and the rest, 29.4% (*n* = 5), were teaching rural hospitals. The median bed capacity (i.e. size) of the hospitals was 150 beds; the largest hospital had 1100 beds, and the smallest hospital had 50 beds. Most of the hospitals (*n* = 10) were private, and the rest were public hospitals (*n* = 7). Most, 82.4% (*n* = 14), were accredited hospitals, and the rest, 52.9% (*n* = 9), were corporate-affiliated hospitals (i.e. part of a hospital network). Hospital characteristics are shown in Table 1.

**Table 1.** A descriptive analysis of hospital characteristics (*N* = 17^a^).

Hospital characteristics	Frequency	Percentage
Hospital location	*n*	%
Urban	12	70.6
Rural	5	29.4
Teaching status	*n*	%
Nonteaching	12	70.6
Teaching	5	29.4
Hospital ownership	*n*	%
Private	10	58.8
Public	7	41.2
Accreditation status	*n*	%
Accredited	14	82.4
Non-accredited	3	17.6
Hospital affiliation	*n*	%
Corporate (part of hospital network)	9	52.9
Stand-alone hospital	8	47.1
Hospital size	Median	IQR
Bed capacity	150	225

IQR, inter-quartile range.

a The number of the included hospitals is 17 (*N* = 17).

A descriptive analysis (mean and standard deviation) of the annual ratings of PREMs and the clinical outcomes is displayed in Table 2. The results indicated that the means for PREMs, LOS, CAUTI, and SSI had improved over the years, while these were fluctuating for hospital mortality and CLABSI.

**Table 2.** A descriptive analysis for the included quality measures across the analysed years (*N* = 221^a^).

Quality measures	2019 (mean, SD)	2020 (mean, SD)	2021 (mean, SD)	2022 (mean, SD)
Number of observations	*n* = 68^a^	*n* = 68^a^	*n* = 68^a^	*n* = 17^a^
PREMs (%)	82.87 (4.82)	84.64 (4.30)	86.64 (3.90)	87.40 (3.88)
Mortality (%)	2.62 (0.83)	2.40 (0.91)	2.50 (0.83)	2.52 (1.01)
Readmission (%)	1.02 (0.65)	0.63 (0.45)	0.62 (0.58)	0.60 (0.74)
LOS (number of days)	4.51 (1.48)	4.20 (1.70)	3.51 (1.78)	3.33 (1.91)
CLABSI rate (per 1000 central line days)	3.71 (1.50)	3.06 (1.57)	3.52 (1.58)	3.91 (2.23)
CAUTI rate (per 1000 catheter days)	2.23 (1.08)	2.01 (1.08)	1.71 (1.02)	1.63 (1.67)
SSI (%)	1.10 (0.68)	0.83 (0.70)	0.77 (0.64)	0.51 (0.48)

a The data matrix included 221 observations (*N* = 221). Each of the included 17 hospitals had four observations every year measured in four consecutive quarters (*n* = 4 * 17 = 68) for the years of 2019, 2020, and 2021. One observation was measured in the year of 2022 as one-quarter only was included (*n* = 1 * 17 = 17).

Bivariate Spearman’s rho correlation tests (Table 3) showed that PREMs had a negative correlation with hospital readmission rate (*r* = −0.332, *P *≤ .01), LOS (*r* = −0.299, *P *≤ .01), CLABSI (*r* = −0.297, *P *≤ .01), CAUTI (*r* = −0.393, *P *≤ .01), and SSI (*r* = −0.298, *P *≤ .01). Patient experience was better in hospitals that had lower rates of readmission and HAIs and shorter LOS. However, no significant correlation was reported between PREMs and hospital mortality. Furthermore, a positive correlation was found between PREMs and hospitals’ teaching status (*r* = 0.196, *P *≤ .01), corporate hospital affiliation (*r* = 0.241, *P *≤ .01), and hospital size (*r* = 0.238, *P *≤ .01), while a negative correlation was reported between PREMs and rural hospital location (*r* = −0.240, *P *≤ .01), indicating better PREM ratings in urban hospitals.

**Table 3.** Bivariate Spearman’s rho correlation tests between the quality measures and hospital characteristics (*N* = 221^a^).

Quality measures	PREMs	Mortality	Readmission	LOS	CLABSI	CAUTI	SSI	Hospital location	Teaching status	Hospital ownership	Accreditation status	Hospital affiliation
PREMs	1.000											
Mortality	−0.068											
Readmission	−0.332**	0.305**										
LOS	−0.299**	−0.074	0.194**									
CLABSI	−0.297**	0.115	0.221**	0.085								
CAUTI	−0.393**	−0.031	0.120	0.210**	0.268**							
SSI	−0.298**	0.434**	0.571**	−0.058	0.177**	0.188**						
Hospital location	−0.240**	0.222**	0.244**	−0.354**	0.075	0.076	0.409**					
Teaching status	0.196**	0.125	0.172*	0.270**	0.197**	0.048	0.033	−0.417**				
Hospital ownership	0.060	0.340**	0.382**	−0.112	−0.010	−0.163*	0.412**	0.509**	−0.015			
Accreditation status	−0.167*	−0.150*	−0.041	0.389**	0.153*	0.053	−0.109	−0.717**	0.299**	−0.553**		
Hospital affiliation	0.241**	−0.213**	−0.307**	0.177**	−0.302**	0.101	−0.483**	−0.167*	0.091	−0.169*	−0.127	
Hospital size	0.238**	−0.094	−0.177**	0.504**	0.032	−0.034	−0.325**	−0.773**	0.667**	−0.296**	0.621**	0.329**

a The data matrix included 221 observations (*N* = 221). Each of the included 17 hospitals had 13 observations measured in 13 consecutive quarters starting from the first quarter of 2019 to the end of the first quarter of 2022 (17 * 13 = 221 observations).

* Correlation is significant at the 0.05 level (two-tailed).

** Correlation is significant at the 0.01 level (two-tailed).

The block-wise modelling multivariate generalized linear mixed model regression analysis was conducted using the full model that included the six clinical outcomes and hospital characteristics included in this study as fixed effects. However, this model was not preferred based on the AIC and BIC values. Accordingly, another model was chosen, including less number of variables by dismissing mortality, readmission, CLABSI, hospital location, ownership, affiliation, and accreditation status from the analysis model. This model performed the best compared to all models analysed in terms of the lower AIC and BIC values. Table 4 includes the multivariate generalized linear mixed model that was finally chosen. The results in Table 4 indicate that PREM scores during the years of 2020 (*β *= 1.181, *P *= .003), 2021 (*β* = 2.443, *P *< .001), and 2022 (*β* = 2.789, *P *< .001) were higher than PREM scores during 2019. In addition, CAUTI and LOS had converged negatively with PREMs (*β* = −0.548, *P *= .005; *β* = −0.873, *P *= .008, respectively). Moreover, our regression model showed that larger hospitals tended to have better PREM scores (*β* =0.009, *P *= .003).

**Table 4.** A multivariate generalized linear mixed regression analysis of PREMs^a^ (*N* = 221^b^).

		95% confidence interval		
	*β* coefficient (SE)	Lower	Upper	*P*-value
Parameter				
Intercept	86.690 (1.911)	82.923	90.458	<.001
Year 2019	Reference			
Year 2020	1.181 (0.413)	0.367	1.994	.003
Year 2021	2.443 (0.649)	1.163	3.723	<.001
Year 2022	2.789 (0.704)	1.401	4.177	<.001
SSI (%)	−0.656 (0.410)	−1.1464	0.152	.111
CAUTI rate (per 1000 catheter days)	−0.548 (0.195)	−0.933	−0.164	.005
LOS (number of days)	−0.873 (0.329)	−1.521	−0.226	.008
Teaching hospital (no)	Reference			
Teaching hospital (yes)	0.304 (1.193)	−2.048	2.657	.799
Hospital size (number of beds)	0.009 (0.003)	0.003	0.015	.003
Model summary^c^				
AIC	1036.865			
BIC	1,053.306			

a The dependent variable is the PREM score (%).

b The data matrix included 221 observations (*N* = 221). Each of the included 17 hospitals had 13 observations measured in 13 consecutive quarters starting from the first quarter of 2019 to the end of the first quarter of 2022 (17 * 13 = 221 observations).

c The goodness-of-fit of the analysis models was evaluated by the AIC and BIC. The model with the lower AIC and BIC values was preferred.

## Discussion

### Statement of principal findings

Our observational study indicated an inverse correlation between PREMs and unfavourable clinical outcomes, including hospital readmission rate, LOS, and HAIs (CLABSI, CAUTI, and SSI) after controlling for hospital characteristics and year in 17 hospitals in Saudi Arabia. Patient experience was better in hospitals that had a better quality of care in terms of fewer readmissions, shorter LOS, and lower infection rates. In addition, hospitals with lower CAUTI and SSI and of larger sizes were associated with better patient experience reports.

### Strengths and limitations

To our knowledge, this is the first study to assess the association between PREMs and the quality of hospital care in Saudi Arabia. Our work is in line with the international efforts to incorporate patient-reported outcomes and experiences to evaluate the quality of care, and to use them to promote shared decision-making with patients and families. The associations reported in this study suggest that hospital quality improvement efforts should go hand in hand with implementing VBHC and patient-centred care initiatives. However, we acknowledge certain limitations in this study. First, our study used a retrospective observational design that cannot identify a causal relationship between PREMs and clinical outcomes. However, strong associations were reported. Second, although our sample size was not as large as sample sizes in other studies, our findings were comparable to other relevant analyses conducted in various countries. Furthermore, we used data collected over an extended period of time to ensure that more data points were included in the analysis. Also, the diversity of the included hospitals was considered in terms of hospital sizes, geographical locations, system affiliations, teaching status, and ownership, enhancing the possibility of generalizing the findings to other settings. Third, due to the retrospective nature of this study, data were missing for some measures. However, the missing values were minimal (<5%), and the values of the variables in the whole dataset were homogenous, suggesting that the values of the missing measures are perhaps consistent with the data included in the analysis. Finally, data analysis was conducted at the hospital level using the overall score of PREMs. More research is needed to further assess associations at the patient level that may provide deeper and more detailed insights into the association between PREMs and clinical outcomes.

### Interpretation within the context of the wider literature

Although there is a lack of clarity in the literature on the association between PREMs and clinical outcomes [7–24], our findings were consistent with previous studies conducted in the USA and Canada that reported an association between patient perceptions of care and performance in terms of hospital readmission [8, 10, 11, 16], CLABSI [11, 13, 14], CAUTI [14], and SSI [14]. These results indicated that PREMs could be considered as an indicator that reflects clinical outcomes experienced in hospitals. In addition, hospitals may implement quality improvement strategies that have dual aims of improving clinical outcomes (e.g. reducing readmission rates and HAIs) and, at the same time, improving PREMs and patient perceptions of care in general.

It is important to note that the data in our study did not enable us to identify a causal relationship. This is relevant because clinical outcomes are multifaceted compared to process measures. Mortality, readmission, LOS, and HAIs can be affected by multiple factors that are not necessarily under the control of the health-care providers, such as sociodemographic factors. It is difficult to solely link improvement in the delivery of health-care services to improvements in these clinical outcomes and, accordingly, better patient experience scores [10]. Thus, although an association was found between the quality measures included in our study, further research is needed to study the impact of the initiatives undertaken to improve the clinical outcomes on patient experience reports. This is important to guide hospitals’ improvement efforts to mutually enhance the quality of care and patient experience at the same time.

Moreover, in congruence with a previous study [7], we found that patients treated in larger hospitals had better patient experience compared to patients in smaller hospitals. An explanation of this finding could be that larger hospitals may have more resources, facilities, diverse specialities, and nationalities of health-care providers that better satisfy patient needs, values, and preferences, ending up with better PREM scores. Furthermore, in our regression model, CAUTI and LOS were associated with PREM scores. Having lower CAUTI rates and shorter LOS had a stronger association with patient experience reports. On the other hand, no significant association between PREMs and mortality was reported in our study. Compared to hospital readmission, LOS, and HAIs, mortality can be influenced by a wide range of factors that are not within the control of health-care providers and institutions. Accordingly, hospitals with higher PREMs are not necessarily associated with lower mortality rates. Future studies may build on our findings and investigate the associations between PREMs and risk-adjusted mortality rates.

### Implications for policy, practice, and research

Given the fact that PREMs are incorporated in international hospital reimbursement systems and knowing that improvements in clinical outcomes are reflected in better patient experiences, our finding is important to the health-care managers, administrators, and policy makers who plan quality improvement initiatives to concurrently improve clinical quality and patient experience. In particular, our results can guide health-care systems that are still in the early stages of transformation to VBHC, like the Saudi health-care system, towards a better understanding of VBHC-related quality measures. In addition, our findings provide assurance to the health-care providers and clinicians that their efforts in improving the quality of care are adding value, are being recognized by the patients, and are reflected in better patient experience reports. However, further research should be conducted to better understand the effect of various quality improvement initiatives on clinical outcomes and PREMs in a hospital setting.

## Conclusion

Implementing VBHC reforms encompasses a wide range of measurement and improvement efforts within the entire health-care system to prioritize what the patients value most. Our findings suggest that in Saudi Arabia, better performance in clinical outcomes is associated with higher PREM scores. Policy makers and administrators in Saudi Arabia and other similar health-care systems can incorporate PREMs in their VBHC programmes to ensure the inclusion of patients’ feedback and input in reforming the health-care systems considering what matters to the patients as reported by the patients themselves. PREMs may not substitute and are not surrogate measures for clinical quality, yet PREMs are complementary when combined with other objective measures of patient-reported outcomes, process of care, and clinical outcomes.

## Supplementary Material

mzad047_Supp

## Data Availability

Data underlying the findings of this study are available within the article [and/or] its supplemental data. Additional data will be shared at reasonable request to the corresponding author in a de-identified form for confidentiality purposes.

## References

[1] Agency for Healthcare Research and Quality (AHRQ) . What Is Patient Experience ? 2016. https://www.ahrq.gov/cahps/about-cahps/patient-experience/index.html (1 February 2023, date last accessed).

[2] Rathert C, Wyrwich M, Boren S. Patient-centered care and outcomes: a systematic review of the literature. *Med Care Res Rev* 2013;70:351–79. 10.1177/1077558712465774. 23169897

[3] Porter M . What is value in health care? *N Engl J Med* 2010;363:2477–81. 10.1056/NEJMp1011024. 21142528

[4] Institute of Medicine (US) Committee on Quality of Health Care in America . *Crossing the Quality Chasm: A New Health System for the twenty-first Century*. Washington, DC: National Academies Press, 2001. 25057539

[5] Berwick D, Nolan T, Whittington J. The triple aim: care, health, and cost. *Health Aff (Millwood)* 2008;27:759–69. 10.1377/hlthaff.27.3.759. 18474969

[6] Centers for Medicare & Medicaid Services (CMS) . The Hospital Value-Based Purchasing (VBP) Program. 2013; https://www.cms.gov/Medicare/Quality-Initiatives-Patient-Assessment-Instruments/Value-Based-Programs/HVBP/Hospital-Value-Based-Purchasing (26 January 2023, date last accessed).

[7] Sacks G, Lawson E, Dawes A et al. Relationship between hospital performance on a patient satisfaction survey and surgical quality. *JAMA Surg* 2015;150:858–64. 10.1001/jamasurg.2015.1108. 26108091

[8] Tsai T, Orav E, Jha A. Patient satisfaction and quality of surgical care in US hospitals. *Ann Surg* 2015;261:2–8. 10.1097/SLA.0000000000000765. 24887985 PMC4248016

[9] Tajeu G, Kazle A, Menachemi N. Do hospitals that do the right thing have more satisfied patients? *Health Care Manage Rev* 2015;40:348–55. 10.1097/HMR.0000000000000034. 26352400

[10] Prabhu K, Cleghorn M, Elnahas A et al. Is quality important to our patients? The relationship between surgical outcomes and patient satisfaction. *BMJ Qual Saf* 2018;27:48–52. 10.1136/bmjqs-2017-00707. 29101291

[11] Kennedy G, Tevis S, Kent K. Is there a relationship between patient satisfaction and favorable outcomes? *Ann Surg* 2014;260:592–600. 10.1097/SLA.0000000000000932. 25203875 PMC4159721

[12] Betts D, Balan-Cohen A. Value of patient experience: hospitals with higher patient experience scores have higher clinical quality. Deloitte Center for Health Solutions, 2017.

[13] Trzeciak S, Gaughan J, Bosire J et al. Association between medicare summary star ratings for patient experience and clinical outcomes in US hospitals. *J Patient Exp* 2016;3:6–9. 10.1177/2374373516636681. 28725825 PMC5513621

[14] Stein S, Day M, Karia R et al. Patients’ perceptions of care are associated with quality of hospital care: a survey of 4605 hospitals. *Am J Med Qual* 2015;30:382–8. 10.1177/1062860614530773. 24740016

[15] Saman D, Kavanagh K, Johnson B et al. Can in-patient hospital experiences predict central line-associated bloodstream infections? *PLoS One* 2013;8:e61097. 10.1371/journal.pone.0061097. PMC3618432 23577195

[16] Carter J, Ward C, Wexler D et al. The association between patient experience factors and likelihood of 30-day readmission: a prospective cohort study. *BMJ Qual Saf* 2018;27:683–90. 10.1136/bmjqs-2017-007184. 29146680

[17] Doyle C, Lennox L, Bell D. A systematic review of evidence on the links between patient experience and clinical safety and effectiveness. *BMJ Open* 2013;3:e001570. 10.1136/bmjopen-2012-001570. PMC3549241 23293244

[18] Sheetz K, Waits S, Girotti M et al. Patients’ perspectives of care and surgical outcomes in Michigan: an analysis using the CAHPS hospital survey. *Ann Surg* 2014;260:5–9. 10.1097/SLA.0000000000000626. 24646549 PMC4049268

[19] Black N, Varaganum M, Hutchings A. Relationship between patient reported experience (PREMs) and patient reported outcomes (PROMs) in elective surgery. *BMJ Qual Saf* 2014;23:534–42. 10.1136/bmjqs-2013-002707. 24508681

[20] Day M, Hutzler L, Karia R et al. Hospital-acquired conditions after orthopedic surgery do not affect patient satisfaction scores. *J Healthc Qual* 2014;36:33–40. 10.1111/jhq.12031. 24033917

[21] Levin J, Winkelman RD, Smith G et al. The association between the Hospital Consumer Assessment of Healthcare Providers and Systems (HCAHPS) survey and real-world clinical outcomes in lumbar spine surgery. *Spine J* 2017;17:1586–93. 10.1016/j.spinee.2017.05.002. 28495242

[22] Lyu H, Wick E, Housman M et al. Patient satisfaction as a possible indicator of quality surgical care. *JAMA Surg* 2013;148:362–7. 10.1001/2013.jamasurg.27. 23715968

[23] Prang K, Canaway R, Bismark M et al. Associations between patient experiences and clinical outcomes: a cross-sectional data linkage study of the Australian private healthcare sector. *BMJ Open Quality* 2019;8:e000637. 10.1136/bmjoq-2019-000637. PMC6711428 31523739

[24] Gupta D, Rodeghier M, Lis C. Patient satisfaction with service quality as a predictor of survival outcomes in breast cancer. *Support Care Cancer* 2014;22:129–34. 10.1007/s00520-013-1956-7. 24013568

[25] Ministry of Health, Saudi Arabia . Patient Satisfaction 2020. https://www.moh.gov.sa/Pages/Default.aspx (12 February 2023, date last accessed).

[26] Senitan M, Alhaiti A, Gillespie J. Patient satisfaction and experience of primary care in Saudi Arabia: a systematic review. *Int J Qual Health Care* 2018;30:751–9. 10.1093/intqhc/mzy104. 29860320

[27] Senitan M, Gillespie J. Healthcare reform in Saudi Arabia: patient experience at primary healthcare centers. *J Patient Exp* 2020;7:587–92. 10.1177/2374373519872420. 33062882 PMC7534112

[28] Bolfotouh M, Al-Assiri M, Alshahrani R et al. Predictors of patient satisfaction in an emergency care centre in central Saudi Arabia: a prospective study. *Emerg Med J* 2017;34:27–33. 10.1136/emermed-2015-20495. 27480456 PMC5256124

[29] Banaser M, Stoddart K, Cunningham N. A qualitative study of patient satisfaction in oncology wards setting in Saudi Arabia. *Res Rev* 2017;3:85–97.

[30] Malott D, Fulton B, Rigamonti D et al. Psychometric testing of a measure of patient experience in Saudi Arabia and the United Arab Emirates. *J Surv Stat Methodol* 2017;5:398–408. 10.1093/jssam/smx008.

