# Supplementary material for: Association of patient experience and the quality of hospital care
Source: Int J Qual Health Care. 2023 Jul 3;35(3):mzad047. doi: 10.1093/intqhc/mzad047 (PMC10321378; doi:10.1093/intqhc/mzad047)
Supplement: mzad047_Supp [file mzad047_supp.zip › suppl_data/Supplementary File 1.docx]

**Supplementary File 1: Summary of the relevant literature on the association of patient experience and the quality of hospital care.**

| Reference Number ^1^ | First Author (Year) | Country | Design | Sample Size | Included Quality Measures | Conclusion |
| --- | --- | --- | --- | --- | --- | --- |
| Studies that show association. | | | | | | |
| 7 | Sacks (2015) | USA | Retrospective observational study | 180 hospitals | Patient satisfaction; thirty-day postoperative mortality; major and minor complications; failure to rescue; and hospital readmission. | A significant association was demonstrated between higher patient satisfaction scores and lower mortality, failure to rescue, and minor complications. |
| 8 | Tsai (2015) | USA | Retrospective observational study | 2953 hospitals | Patient satisfaction; risk-adjusted length of stay; Surgical Care Improvement Project (SCIP) score; risk-adjusted mortality rate; risk-adjusted readmission rate. | An association was found between higher patient satisfaction and shorter length of stay, higher surgical process scores, and lower mortality and readmission rate. |
| 9 | Tajeu (2015) | USA | Retrospective observational study | 13,997 hospital-year observations | Patient satisfaction, acute myocardial infarction, heart failure, and pneumonia. | A positive association was found between patient satisfaction and measures of acute myocardial infarction, heart failure, and pneumonia. |
| 10 | Prabhu (2018) | USA | Retrospective observational study | 757 patients | Patient satisfaction; postoperative surgical complications. | A significant association was found between patient satisfaction and both 30-day readmission and the occurrence of postoperative surgical complications. |
| 11 | Kennedy (2014) | USA | Retrospective observational study | 171 hospitals | Patient satisfaction; Surgical Care Improvement Project (SCIP) score; length of stay; complications; mortality; patient safety indicators including the adverse postoperative events. | Hospital size, surgical volume and low mortality were associated with high overall patient satisfaction. However, with the exception of low mortality, favorable surgical outcomes were not consistently associated with high patient satisfaction scores. |
| 12 | Betts (2017) | USA | Retrospective observational study | -- | Patient experience; ED timeliness of care; surgical process of care; preventive care; information/education; effectiveness of care; mortality; readmissions; Hospital Acquired Infections (HAI). | Hospitals with higher patient reported experience ratings have better process of care quality scores and better scores for some, but not all, clinical outcomes. |
| 13 | Trzeciak (2016) | USA | Retrospective observational study | > 3000 hospitals | Patient experience; hospital complications (central line-associated bloodstream infection CLABSI, postsurgical deep venous thrombosis, joint replacement complications, and a composite measure for all serious complications); unplanned readmissions within 30 days of discharge. | Better patient experience was associated with favorable clinical outcomes including lower rates of hospital complications and readmission. |
| 14 | Stein (2015) | USA | Retrospective observational study | 4605 hospitals | Patient experience; complication rate. | An inverse relationship was reported between patient experience and complication rates. |
| 15 | Saman (2013) | USA | Retrospective observational study | 1987 hospitals | Patient experience; CLABSI in Intensive Care Units (ICUs). | Lower inpatients’ hospital experiences were significantly associated with an increased risk of ICU reported CLABSIs. |
| 16 | Carter (2018) | USA | Prospective cohort study | 846 patients | Patient satisfaction; 30-day readmission. | Participants reporting high satisfaction and good provider communication were less likely to be readmitted. |
| 17 | Doyle (2013) | -- | Systematic review | 55 studies | Patient experience; a broad range of patient safety and clinical effectiveness outcomes including mortality, physical symptoms, length of stay and adherence to treatment. | Patient experience was positively associated with clinical effectiveness and patient safety and support the case for the inclusion of patient experience as one of the central pillars of quality in healthcare. |
| -- | Kemp (2016) ^2^ | Canada | Cross-sectional study | 93 hospitals | Patient experience; patient safety indicators. | An association was found between higher inpatient experience ratings and lower adverse events as documented by patient safety indicators. |
| -- | Diwan (2020) ^3^ | USA | A retrospective observational study | 600 patients | Patient satisfaction; length of stay. | Increased length of stay is associated with lower patient satisfaction and decreased likelihood of recommending the hospital. |
| Studies that show no or low association. | | | | | | |
| 18 | Sheetz (2014) | USA | Retrospective observational study | 41,833 patients | Patient experience; risk-adjusted postoperative morbidity and mortality. | Patients' perspectives of care do not correlate with the incidence of morbidity and mortality following major surgery. |
| 19 | Black (2014) | England | Cross-sectional study | 10383 patients | Patient experience; patient-reported outcome measures on effectiveness and safety. | A weak positive association between experience and effectiveness for all three procedures (hip replacement, knee replacements, and groin hernia repairs). |
| 20 | Day (2014) | USA | Cross-sectional study | 6,056 patients | Patient satisfaction; surgical site infection; venous thromboembolism. | Development of hospital acquired conditions was not associated with decreased satisfaction scores in a population of orthopedic surgery patients at a private, university-affiliated specialty center. |
| 21 | Levin (2017) | USA | A retrospective observational study | 249 patients | Patient satisfaction; self-reported health status measures. | Higher patient satisfaction rating was not associated with better self-reported health status measures. |
| 22 | Lyu (2013) | USA | Retrospective observational study | 31 hospitals | Patient satisfaction; Surgical Care Improvement Program compliance score; hospital employee safety attitudes (safety culture). | Patient satisfaction was independent of hospital compliance with surgical processes of quality care and with overall hospital employee safety culture, although a few individual domains of culture were associated |
| 23 | Prang (2019) | Australia | Cross-sectional study | 692 hospitals | Patient experience; 14-day readmission; 28-day readmission; hospital-acquired complications; length of stay. | Absence of many associations was reported between patient experience domains and clinical outcomes suggesting that patient experiences should not be viewed as a surrogate marker of good clinical outcomes. |
| 24 | Gupta (2014) | USA | Cross-sectional study | 1,521 patients | Patient satisfaction; survival in breast cancer patients. | Patient satisfaction with service quality was an independent predictor of survival in breast cancer. |

^1^ The reference number was taken from the reference list in the article.

^2^ Kemp K, Santana M, Southern *D et al*. Association of inpatient hospital experience with patient safety indicators: a cross-sectional, Canadian study. *BMJ Open* 2016;6(7):e011242.

^3^ Diwan W, Nakonezny P, Wells J. The Effect of Length of Hospital Stay and Patient Factors on Patient Satisfaction in an Academic Hospital. *Orthopedics* 2020;43(6):373-379.
